# Evaluation of Multiplex Rapid Antigen Tests for the Simultaneous Detection of SARS-CoV-2 and Influenza A/B Viruses

**DOI:** 10.3390/biomedicines11123267

**Published:** 2023-12-09

**Authors:** Ho-Jae Lim, Ji-Yoon Lee, Young-Hyun Baek, Min-Young Park, Dong-Jae Youm, Inhee Kim, Min-Jin Kim, Jongmun Choi, Yong-Hak Sohn, Jung-Eun Park, Yong-Jin Yang

**Affiliations:** 1 Department of Molecular Diagnostics, Seegene Medical Foundation, Seoul 04805, Republic of Korea; 52rotc.hjl@mf.seegene.com (H.-J.L.); yiy2190@mf.seegene.com (J.-Y.L.); baek0h@mf.seegene.com (Y.-H.B.); pyli186@mf.seegene.com (M.-Y.P.); ehdwo01@mf.seegene.com (D.-J.Y.); ihkim@mf.seegene.com (I.K.); lithium2864@mf.seegene.com (M.-J.K.); fsysy@mf.seegene.com (J.C.); medsohn@mf.seegene.com (Y.-H.S.); 2 Department of Integrative Biological Sciences & BK21 FOUR Educational Research Group for Age-Associated Disorder Control Technology, Chosun University, Gwangju 61452, Republic of Korea

**Keywords:** rapid antigen test, rRT-PCR, SARS-CoV-2, influenza A virus, influenza B virus, cut-off, multiplex tests, SARS-CoV-2 variant

## Abstract

Single-target rapid antigen tests (RATs) are commonly used to detect highly transmissible respiratory viruses (RVs), such as severe acute respiratory syndrome coronavirus 2 (SARS-CoV-2) and influenza viruses. The simultaneous detection of RVs presenting overlapping symptoms is vital in making appropriate decisions about treatment, isolation, and resource utilization; however, few studies have evaluated multiplex RATs for SARS-CoV-2 and other RVs. We assessed the diagnostic performance of multiplex RATs targeting both the SARS-CoV-2 and influenza A/B viruses with the GenBody Influenza/COVID-19 Ag Triple, InstaView COVID-19/Flu Ag Combo (InstaView), STANDARD^TM^ Q COVID-19 Ag Test, and STANDARD^TM^ Q Influenza A/B Test kits using 974 nasopharyngeal swab samples. The cycle threshold values obtained from the real-time reverse transcription polymerase chain reaction results showed higher sensitivity (72.7–100%) when the values were below, rather than above, the cut-off values. The InstaView kit exhibited significantly higher positivity rates (80.21% for SARS-CoV-2, 61.75% for influenza A, and 46.15% for influenza B) and cut-off values (25.57 for SARS-CoV-2, 21.19 for influenza A, and 22.35 for influenza B) than the other two kits, and was able to detect SARS-CoV-2 Omicron subvariants. Therefore, the InstaView kit is the best choice for routine screening for both SARS-CoV-2 and influenza A/B in local communities.

## 1. Introduction

Coronavirus disease 2019 (COVID-19), which is caused by severe acute respiratory syndrome coronavirus 2 (SARS-CoV-2) infection, was first reported in December 2019 and rapidly led to a pandemic [1]. Despite concerted global efforts to control the spread of SARS-CoV-2, the successive waves of the virus placed health systems in many countries under enormous pressure [2]. Although vaccination is an effective method of controlling SARS-CoV-2 infection, the emergence of new variants and the lack of long-lasting immunity have resulted in the persistence of the pandemic for almost 4 years [3].

The World Health Organization declared an end to the COVID-19 Public Health Emergency of International Concern on 5 May 2023. This resolution was predicated on the observed decrease in mortality rates and the number of hospitalized cases, in conjunction with high levels of both individual and herd immunity [4]. Nevertheless, SARS-CoV-2 continues to be a global concern because of its potential to evolve; therefore, long-term monitoring is necessary [5]. The management of COVID-19 is hindered by the necessity of distinguishing single SARS-CoV-2 infection from co-infection or infection with other respiratory viruses (RVs), such as influenza virus and non-SARS-CoV-2 coronaviruses [6,7]. In particular, the clinical manifestations of COVID-19 and influenza are similar, thus requiring accurate viral identification [7,8].

Although the incidence of co-infection with SARS-CoV-2 and the influenza virus is relatively low, it is associated with a substantially increased risk of death [7]. Seasonal influenza viruses are also an important global public health threat, and they are highly transmissible through exposure to droplets and aerosol particles, without direct contact [9]. Therefore, rapid and reliable tests are needed to screen for RVs and provide accurate and timely virological diagnoses in patients with signs of viral respiratory infection.

Several categories of tests are currently available for the diagnosis of RV infection, including nucleic acid amplification tests (loop-mediated isothermal amplification and real-time reverse-transcription polymerase chain reaction (rRT-PCR) tests) and immunoassay antigen-based tests. Laboratory-based rRT-PCR assays using nasopharyngeal swab (NPS) specimens are highly sensitive and specific for the detection of RVs [10]. Compared with other diagnostic methods, rRT-PCR assays require a testing time of 3–4 h, owing to the additional steps required, which include RNA isolation and amplification [11]. The long testing times, testing requirements (such as laboratory setting, sample preparation, and result interpretation), and increased testing demand have led to testing bottlenecks that negatively affect patient management [12,13]. This has led to a need for alternative tests, such as highly sensitive rapid antigen tests (RATs), which can be performed at the point of care and can detect multiple pathogens [14,15].

Compared with rRT-PCR assays, RATs are simple to perform and provide test results within minutes. Moreover, RAT results can be read with the naked eye, without the need for expensive laboratory equipment [16]. Previous studies have evaluated the clinical performance of commercially available RAT kits for the detection of a single pathogen (SARS-CoV-2 or influenza A/B viruses). Compared with rRT-PCR methods, the reported sensitivity of RATs varies substantially (53.8–100%) depending on the sample distribution, whereas the specificity of RATs is generally comparable to that of rRT-PCR (81.8–100%) [16,17,18]. Other studies have evaluated the clinical performance of commercially available RAT kits for the simultaneous detection of SARS-CoV-2, influenza A virus (FluA), and influenza B virus (FluB) [19]. However, to date, few studies have evaluated multiplex RATs for SARS-CoV-2 and other RVs.

Several multiplexed RAT kits were recently released for field testing [20,21]. Therefore, this study evaluated the diagnostic performance of three RATs for SARS-CoV-2, namely the GenBody Influenza/COVID-19 Ag Triple kit, the InstaView COVID-19/Flu Ag Combo kit, and the STANDARD^TM^ Q COVID-19 Ag Test kit, as well as a RAT for FluA/FluB, namely the STANDARD^TM^ Q Influenza A/B Test kit, compared with the performance of rRT-PCR based assays for the simultaneous detection of SARS-CoV-2, FluA, and FluB in appropriately stored NPS samples. The four multiplexed RATs could be performed in less than 20 min and verified the current effects of mutations in SARS-CoV-2.

## 2. Materials and Methods

### 2.1. Storage of Clinical Specimens

This study was approved by the Institutional Review Board of the Seegene Medical Foundation (SMF-IRB-2022-037). NPS samples from 974 patients (male, *n* = 515; female, *n* = 459) were preserved in transport medium during the laboratory assessment period, spanning from March 2022 to September 2023. These samples underwent molecular diagnostic assays for the detection of RVs at the Seegene Medical Foundation (Seoul, Republic of Korea). Among the anonymized samples, 494 were classified as either SARS-CoV-2-positive or -negative, and 480 were classified as influenza A/B-positive or -negative. The collected specimens were stored at −80 °C until testing. After thawing, RATs and rRT-PCR tests were conducted simultaneously, without any delay.

### 2.2. Viral RNA Isolation and rRT-PCR Assay

Viral RNA was isolated from the 974 samples using the automated MagNA Pure 96 system (Roche Inc., Basel, Switzerland) [22]. The purified viral RNA was then tested using the Allplex^TM^ SARS-CoV-2/FluA/FluB/RSV Assay kit (Seegene Inc., Seoul, Republic of Korea), which detects the RNA-dependent RNA polymerase, spike (*S*), and nucleocapsid (*N*) genes of SARS-CoV-2, FluA, FluB, and respiratory syncytial virus. The rRT-PCR results were interpreted according to the methods used in a previous study [23]. Positive results were classified into three categories: Ct < 20, 20 ≤ Ct < 25, and 25 ≤ Ct < 40. The Ct value of *N* was used for SARS-CoV-2 statistical analysis. The Ct values of rRT-PCR are inversely proportional to the viral load; thus, a lower Ct value indicates a higher viral load.

### 2.3. Rapid Antigen Test Kits and Experimental Protocol

**Figure 1:**
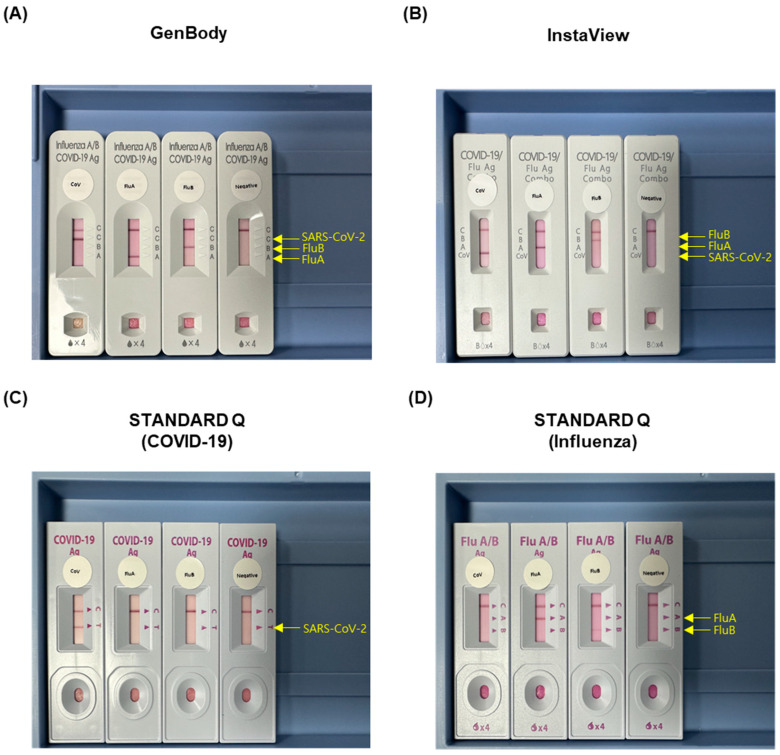
Photographs of the four rapid antigen test kits used. (**A**) GenBody, (**B**) InstaView, (**C**) STANDARD Q (COVID-19), and (**D**) STANDARD Q (Influenza). A band was indicative of a positive result for the labeled antigen (SARS-CoV-2-positive, FluA-positive, and FluB-positive). Abbreviations: FluA, influenza A virus; FluB, influenza B virus; SARS-CoV-2, severe acute respiratory syndrome coronavirus 2.

After extraction, the remaining samples were tested for SARS-CoV-2, FluA, and FluB using four RAT kits: (i) GenBody Influenza/COVID-19 Ag Triple kit (GenBody, GenBody Inc., Cheonan, Republic of Korea); (ii) InstaView COVID-19/Flu Ag Combo kit (InstaView, SG Medical, Seoul, Republic of Korea); (iii) STANDARD^TM^ Q COVID-19 Ag Test kit (SD BIOSENSOR Inc., Suwon, Republic of Korea); and (iv) STANDARD^TM^ Q Influenza A/B Test kit (SD BIOSENSOR Inc.). In this study, the results of the STANDARD Q COVID-19 Ag and Influenza A/B Test kits (iii and iv) were combined. All RAT kits in this study targeted the *N* protein for qualitative viral detection. The RAT procedure was modified from a previously reported procedure [16]. Briefly, 60 μL of transport medium was mixed with 60 μL of the buffer enclosed in each kit. The mixture (1:1 ratio) was then dropped into the sample window of the test device, in accordance with the manufacturer’s instructions. The RAT kits were stored at 20–22 °C for 15–20 min before analysis. The results obtained from the four RAT kits were analyzed by two authors (J.-Y.L. and Y.-H.B.). After dropping the appropriate volume of sample into the well, one visible band should be consistently displayed as the control line, whereas the presence of another band at the SARS-CoV-2, FluA, or FluB site was interpreted as a positive result (Figure 1). In the event of discrepant interpretations, another author (H.-J.L.) was consulted to make the final decision. During the analysis, the results of the STANDARD^TM^ Q COVID-19 Ag Test kit for SARS-CoV-2 and the STANDARD^TM^ Q Influenza A/B Test kit for FluA and FluB were combined (STANDARD Q) and subsequently compared with the results of the GenBody and InstaView multiplex kits.

### 2.4. Next-Generation Sequencing (NGS) and Phylogenetic Analysis of SARS-CoV-2 Variants

NGS was conducted on a randomly selected set of 32.2% (100/311) of SARS-CoV-2-positive samples in both the rRT-PCR and RAT kit results, using the NextSeq 2000 platforms (Illumina, San Diego, CA, USA). The NGS procedure was performed using a validated method, as reported previously [24]. Briefly, viral RNA was amplified with SARS-CoV-2 Emerging Variants Panel Add-on v2 (Paragon Genomics, Hayward, CA, USA). The FASTQ files were processed by cloud analysis using the Flomics pipeline (Flomics, Barcelona, Spain) based on the Wuhan-CoV reference sequence (NCBI accession ID: NC_045512.2). The total number of reads for mapping, the % of mapped reads, and the depth are summarized in Supplementary Table S1. SARS-CoV-2 Omicron sublineages were assigned using the Pangolin Tool (ver.4.3, https://cov-lineages.org/resources/pangolin.html, accessed on 26 September 2023) based on the presence of mutations. Phylogenetic analysis was conducted after removing low-quality bases and artifact sequences. The NCCP-43330 strain was used for the B lineage (the original haplotype of the pandemic) and a clinical sample for the BA.1 lineage (Omicron), which were aligned to the reference genome using the Burrow–Wheeler Aligner (BWA v.0.7.17) [25]. Subsequently, SNPs in our sample set were called by the “HaplotypeCaller” of GATK (v.4.3.0.0) [26]. To avoid false-positive calls, we applied “VariantFiltration” in GATK using the parameters recommended by the tool. Finally, SNPs with missing genotype rates > 0.05 and non-biallelic SNPs were filtered out, resulting in the retention of high-quality variants. Using these high-quality variants, a maximum likelihood method with IQ-TREE (v.2.2.03), ModelFinder, ultrafast bootstrap (1000 replicates), and tree reconstruction options was applied [27,28,29]. According to the Bayesian Information Criterion, the best-fit model was K3Pu (three substitution types model and unequal base frequency) + F (empirical codon frequencies).

### 2.5. Statistical Analysis

All graphs and data analyses excluding phylogenetic analysis were performed using the R software version 4.2.2 (R Foundation for Statistical Computing, Vienna, Austria). Sequence alignment and phylogenetic tree construction were performed using the Python software version 3.10.9 (Python Software Foundation, Wilmington, DE, USA). The receiver operating characteristic (ROC) curves were produced using the “ROCR” and “pROC” packages [30], the scatterplots were produced using the “ggplot2” and “ggbeeswarm” packages, and the survival curves were generated using the “Survival” and “SurvMiner” packages [31]. The optimal cut-off values were estimated using the Youden index, based on the ROC curve, enabling the classification of rRT-PCR-positive and RAT-positive/-negative specimens, as defined by the area under the curve (AUC) [32]. Dot plots were used to compare the differences between the cut-off values from the rRT-PCR and RAT kits. The significance of differences between the rRT-PCR Ct values and the results of each RAT assay was determined using the log-rank test. *p*-values ≤ 0.05 were considered statistically significant.

## 3. Results

### 3.1. Performance of a rRT-PCR Assay Using Frozen–Thawed Samples

A total of 974 NPS specimens were evaluated in this study, of which 903 samples were positive for at least one of the viruses tested, including 485 that were SARS-CoV-2-positive, 400 that were FluA-positive, and 26 that were FluB-positive. Of these, viral co-infection was identified in eight samples (SARS-CoV-2/FluA, *n* = 6; SARS-CoV-2/FluB, *n* = 1; FluA/FluB, *n* = 1). Positive samples were categorized according to their Ct values (Table 1). Overall, the ratios of the Ct values for SARS-CoV-2, FluA, and FluB were similar within each group, except for a few cases in which SARS-CoV-2 had Ct values between 20 and 25, whereas FluA had Ct values between 25 and 40.

### 3.2. Comparison of the Positivity of the RAT Kits

**Figure 2:**
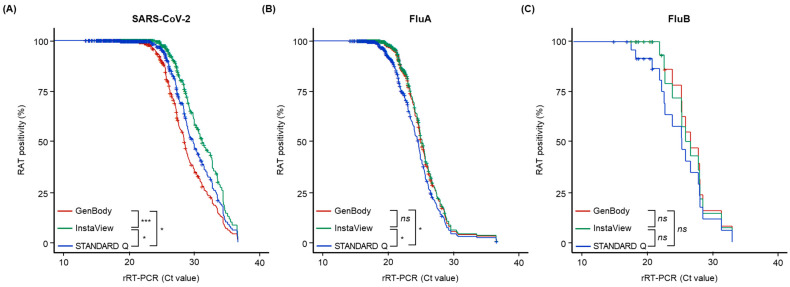
Relationship between the rapid antigen test (RAT) positivity and the cycle threshold (Ct) values, as determined by rRT-PCR assays, with curves showing the diagnostic performance of these two methods in detecting (**A**) SARS-CoV-2, (**B**) FluA, and (**C**) FluB. The colored lines represent the diagnostic performance of each RAT kit: maroon, GenBody; green, InstaView; blue, STANDARD Q. *p*-values indicate the statistical significance of differences between two kits determined using the log-rank test (* *p* ≤ 0.05; *** *p* < 0.001; ns, non-significant, *p* > 0.05). Abbreviations: Ct, cycle threshold; FluA, influenza A virus; FluB, influenza B virus; RAT, rapid antigen test; rRT-PCR, real-time reverse-transcription polymerase chain reaction; SARS-CoV-2, severe acute respiratory syndrome coronavirus 2.

All the RAT kits showed positive results for the control line, without inhibition. The survival analysis, as determined by the minimum Ct value on rRT-PCR required for a negative result using the RAT kits, is shown in Figure 2. For SARS-CoV-2, the minimum Ct values for a RAT-negative result using the GenBody, InstaView, and STANDARD Q kits were 21.27, 24.01, and 18.67, respectively. Using the GenBody, InstaView, and STANDARD Q kits, the minimum Ct values for a RAT-negative result were 18.81, 18.81, and 16.74, respectively, for FluA, and 21.87, 21.87, and 17.46, respectively, for FluB. Of the samples tested using the GenBody, InstaView, and STANDARD Q RAT kits, 261, 338, and 304, respectively, were positive for SARS-CoV-2; 241, 247, and 199, respectively, were positive for FluA; and 13, 12, and 8, respectively, were positive for FluB. The FluB positivity rate did not differ significantly according to the RAT kit type (*p* > 0.05), whereas the SARS-CoV-2 positivity rate differed significantly according to the RAT kit type (*p* < 0.05), and the FluA positivity rate was significantly lower using the STANDARD Q kits than that with the other two kit types (*p* < 0.05).

### 3.3. Diagnostic Performance of the RAT Kits

**Figure 3:**
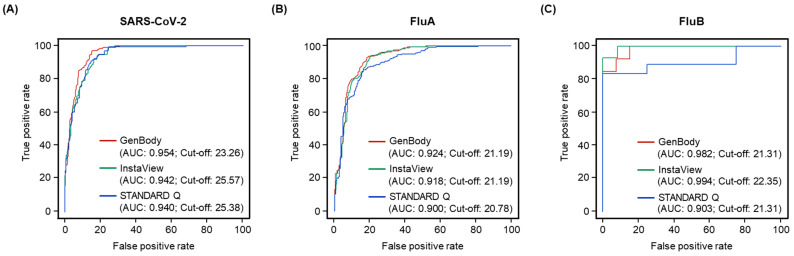
ROC curves showing the diagnostic performance of three RATs based on the Ct values. (**A**) SARS-CoV-2, (**B**) FluA, and (**C**) FluB. The colored lines represent the diagnostic performance of each RAT kit: maroon, GenBody; green, InstaView; blue, STANDARD Q. Abbreviations: AUC, area under the curve; Ct, cycle threshold; FluA, influenza A virus; FluB, influenza B virus; SARS-CoV-2, severe acute respiratory syndrome coronavirus 2.

ROC curves were constructed, and AUCs were calculated to determine the diagnostic accuracy and cut-off values of the RAT kits based on the Youden index (Figure 3). The AUC values ranged from 0.940 to 0.954 for SARS-CoV-2, 0.900 to 0.924 for FluA, and 0.903 to 0.994 for FluB. The cut-off values determined based on the Youden index were 23.26–25.57 for SARS-CoV-2, 20.78–21.19 for FluA, and 21.31–22.35 for FluB. The GenBody RAT provided the highest AUCs for SARS-CoV-2 (0.954) and FluA (0.924), whereas the InstaView RAT provided the highest AUC for FluB (0.994). InstaView had higher cut-off values than the other two kits for SARS-CoV-2 and FluB, whereas the InstaView and GenBody RATs had the same cut-off values for FluA, with both being higher than those of the STANDARD Q kit. Therefore, the GenBody kit had higher AUCs than the other two kits, whereas the InstaView kit had higher cut-off values than the other two kits.

### 3.4. Comparison of RAT Kit Cut-Off Values

Of the 974 NPS samples, 903 tested positive for at least one pathogen as determined by rRT-PCR. Compared with the rRT-PCR assay, regardless of the cut-off values, the assay performed using the RAT kits had sensitivity of 64.33–80.21% for SARS-CoV-2, 49.75–61.75% for FluA, and 30.77–50% for FluB. All three RAT kits had specificity of 100% for the three viruses, except for the GenBody kit, which had specificity of 99.83% for FluA based on one false-positive result (Table 2). A repeated test using the GenBody kit did not reproduce the false-positive result. The kappa value of the RAT kits compared with the rRT-PCR results ranged from 0.48 to 0.80. An analysis stratified by sex showed similar performance in samples from male and female patients (Supplementary Table S2).

**Figure 4:**
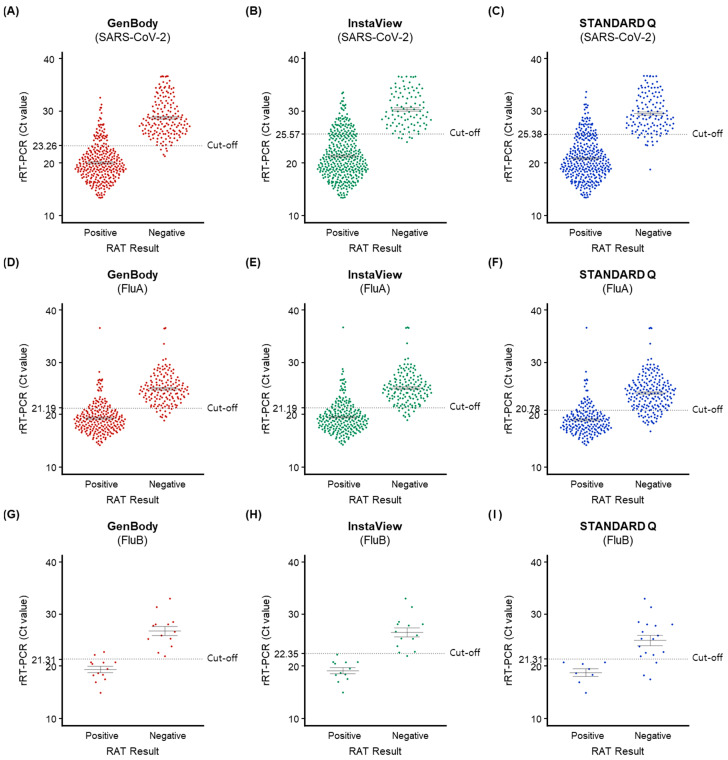
Positivity of the three rapid antigen tests (RATs) according to the cycle threshold (Ct) values of the corresponding rRT-PCR test for the same virus. The dot plots show the positive (left) and negative (right) results for rRT-PCR, and the dotted horizontal line indicates the cut-off values for RAT kits to define a positive result (values below the line are positive and values above the line are negative). (**A**) SARS-CoV-2, GenBody; (**B**) SARS-CoV-2, InstaView; (**C**) SARS-CoV-2, STANDARD Q; (**D**) FluA, GenBody; (**E**) FluA, InstaView; (**F**) FluA, STANDARD Q; (**G**) FluB, GenBody; (**H**) FluB, InstaView; (**I**) FluB, STANDARD Q. Abbreviations: Ct, cycle threshold; FluA, influenza A virus; FluB, influenza B virus; RAT, rapid antigen test; rRT-PCR, real-time reverse-transcription polymerase chain reaction; SARS-CoV-2, severe acute respiratory syndrome coronavirus 2.

As shown in Figure 4, the positivity of samples with Ct values below the cut-off value on rRT-PCR ranged from 96.2% to 98.1%, 85.1% to 95.2%, and 72.7% to 100% for SARS-CoV-2, FluA, and FluB, respectively. The RAT evaluation of samples with Ct values above cut-off values on rRT-PCR showed that 22.2–44.8%, 16.1–25.9%, and 0–13.3% were positive for SARS-CoV-2, FluA, and FluB, respectively. These findings indicate that the diagnostic accuracy of the RAT kits was high when the Ct value was below the cut-off value, and it was low when the Ct value was above the cut-off value.

## 4. Discussion

RVs with the potential for epidemic and pandemic emergence and reemergence continue to pose a threat to human life [33]. RVs can easily spread to multiple individuals through simple contact, and RNA viruses can readily mutate, leading to the emergence of new variants [9,34]. This is illustrated by the emergence of SARS-CoV-2, which led to the COVID-19 pandemic, and epidemic influenza viruses [1,35]. Therefore, continuous monitoring, reliable diagnostic tests, and the simultaneous on-site detection of RV infections are necessary to effectively prevent disease transmission [20].

In previous studies, the sensitivity of RAT kits from three different manufacturers (GenBody Inc., SG Medical, and SD BIOSENSOR Inc.) for SARS-CoV-2 was evaluated using singleplex tests. The sensitivity ranged from 79.9% to 95.6% for GenBody, 93.4% for InstaView, and 55.7% to 98.3% for STANDARD Q [16,36,37,38,39,40,41]. The clinical performance of multiplex RAT kits has been reported to be similar to that of singleplex RAT kits [42]. In this study, we evaluated the sensitivity of three lateral flow-based RATs produced by three different manufacturers (GenBody Inc., SG Medical, and SD BIOSENSOR Inc.). We then compared the performance of the three kits with that of the multiplex rRT-PCR assay. Although the performance of these three RATs varied, the InstaView kit was substantially more sensitive than the other two RATs in detecting SARS-CoV-2, whereas the GenBody and InstaView RATs showed similar performance in detecting FluA. As fewer results were positive for FluB than for SARS-CoV-2 and FluA, the differences in the detection of FluB observed between the three RATs were not statistically significant.

Various factors, such as signal amplification methods, freeze–thaw cycles, and the epitopes of an antigen, affect pathogen identification using RATs [43,44,45]. The signal amplification of RATs can be improved using methods such as chemical enhancement, surface-enhanced Raman scattering, fluorescence, and photo-thermal, electrochemical, and magnetic reactions [43]. The repeated freezing and thawing of stored samples can have an impact on antigen testing, resulting in a 4–5% reduction in analytical sensitivity in samples with a low viral load [44]. Viral mutations can alter the viral epitopes that are recognized by the capturing antibodies. These alterations affect the ability of antibodies to detect antigens and can result in up to a 10-fold reduction in sensitivity [45]. Therefore, in this study, we specifically limited the freeze–thaw cycles to ≤1 and monitored *N* mutations in 32.2% (100/311) of the samples for SARS-CoV-2-positive samples. *N* substitutions (R203K and S413R) were detected in all samples tested. Additionally, 20 other substitutions were detected in a few samples, including P13L, P13F, T24I, S33F, A35T, G96D, E136D, S187L, T165I, T166I, L167F, S187L, R203K, G204P, G204R, L219F, G236S, A267V, D288N, and S413R (Supplementary Figure S1). Among the three RAT kits, Omicron subvariants did not have an impact on the kit performance of any of the three RAT kits.

Generally, the cut-off values for RATs are highly reliable, with Ct values typically below 25 for symptomatic patients [46]. Regardless of the cut-off values, all three RATs exhibited consistently low sensitivity. When detecting SARS-CoV-2, the InstaView and GenBody kits had similar sensitivity below the cut-off values for 98.1% of samples, but, paradoxically, the Ct cut-off values of the InstaView kit were 4.9-fold higher than those of the GenBody kit. Overall, the sensitivity of the InstaView kit was comparable to that of the other RATs, with similar or superior cut-off values. According to the viral shedding pattern, it can be assumed that, following infection with the virus, the viral load increases rapidly during the early stages and then gradually declines [47]. In this study, discrepancies in the sensitivity of the RAT kits were also evident, with the InstaView kit being able to detect higher Ct values, which suggests its potential for slightly earlier detection of infection than that of the other RAT kits.

It has been posited that, in some studies, Ct values may fluctuate because of variability in the sample quality and may not exhibit a consistent correlation with the presence of viral antigens. The diagnostic performance of RAT kits has been reported to vary according to the presence or absence of clinical symptoms [20]. Several reports highlight sex differences (male and female) in clinical symptoms and mortality [48,49]. However, the current study does not suggest a sex-related difference in the performance of RAT kits for the detection of SARS-CoV-2 (Supplementary Table S2). Consequently, future studies should evaluate the performance of RATs by calculating refined cut-off values and using samples from symptomatic individuals, regardless of sex, who have been tested for the presence of SARS-CoV-2, FluA, and FluB. Several studies have demonstrated that multiplex RATs can be useful as a screening tool for the simultaneous detection of pathogens with similar clinical manifestations, offering benefits such as ease of use, cost savings, and a short turnaround time using a single device [50,51].

This study had some limitations. First, we used a universal transport medium containing swabs, rather than directly collected swabs. Although the manufacturers recommended the use of NPS samples, we conducted an indirect evaluation using samples containing NPS. Second, although PCR testing confirmed co-infection in 10 samples, we were unable to evaluate samples with a high viral load that produced multiple bands in the RATs. Lastly, we had no clinical information on the symptoms of the patients at the time of sample collection because we received a random sample of anonymized samples for testing on request. Therefore, further studies should be conducted using fresh NPS samples to confirm these results, and the clinical manifestations should be considered in the interpretation of the results. This study showed that RATs can detect SARS-CoV-2, including SARS-CoV-2 variants, FluA, and FluB, with high diagnostic accuracy, indicating that a positive test result does not require additional confirmatory testing.

## 5. Conclusions

Considering the emergence of multiple, highly transmissible RVs, there is a great need for simultaneous diagnostic methods to mitigate the community transmission of RVs. The RAT kits from the three manufacturers used in this study demonstrated high sensitivity for SARS-CoV-2, FluA, and FluB detection in samples with high viral loads (below the Ct cut-off values on rRT-PCR testing). However, the sensitivity of these kits markedly diminished in samples with low viral loads and Ct values that exceeded Ct cut-off values on rRT-PCR testing, particularly in samples with Ct values above 25. Thus, RAT kits should be used judiciously, given the elevated risk of false-negative results when detecting SARS-CoV-2, FluA, and FluB in samples with low viral loads. Multiplex RAT kits can detect various RVs using a single specimen, without any difference in performance, compared with singleplex RAT kits. Among the evaluated kits, the InstaView kit demonstrated a substantially higher sensitivity than that of the other RAT kits. This suggests its potential value for use as a routine diagnostic tool to screen for RVs in communal environments, especially when high sensitivity in the detection of SARS-CoV-2, FluA, and FluB is important.

## Figures and Tables

**Table 1:** Classification of rRT-PCR results according to the Ct value.

rRT-PCR	Negative	Positive			
		Total	Ct < 20	20 ≤ Ct < 25	25 ≤ Ct < 40
SARS-CoV-2	489	485	175	127	183
FluA	574	400	169	151	80
FluB	948	26	7	9	10

Abbreviations: Ct, cycle threshold; FluA, influenza A virus; FluB, influenza B virus; rRT-PCR, real-time reverse-transcription polymerase chain reaction; SARS-CoV-2, severe acute respiratory syndrome coronavirus 2.

**Table 2:** Comparison of the diagnostic accuracy of rapid antigen test kits using reverse-transcription polymerase chain reaction tests as the comparator.

Pathogens	RAT Kits	TP (*n*)	FP (*n*)	TN (*n*)	FN (*n*)	Se (%)	Sp (%)	Kappa
SARS-CoV-2	GenBody	312	0	489	173	64.33	100	0.64
	InstaView	389	0	489	96	80.21	100	0.80
	STANDARD Q	355	0	489	130	73.2	100	0.73
FluA	GenBody	241	1	573	159	60.25	99.83	0.64
	InstaView	247	0	574	153	61.75	100	0.66
	STANDARD Q	199	0	574	201	49.75	100	0.54
FluB	GenBody	13	0	948	13	50	100	0.66
	InstaView	12	0	948	14	46.15	100	0.63
	STANDARD Q	8	0	948	18	30.77	100	0.48

Abbreviations: FluA, influenza A virus; FluB, influenza B virus; FN, false negative; FP, false positive; RAT, rapid antigen test; SARS-CoV-2, severe acute respiratory syndrome coronavirus 2; Se, sensitivity; Sp, specificity; TN, true negative; TP, true positive.

## Data Availability

The data presented in this study are available on request from the corresponding author.

## Supplementary Materials

The following are available online at https://www.mdpi.com/article/10.3390/biomedicines11123267/s1, Supplementary Table S1: Mapping statistics of derived sequences of SARS-CoV-2 genomes detected using metagenomics next-generation sequencing; Supplementary Table S2: Diagnostic accuracy of RAT kits compared with rRT-PCR according to sex; Supplementary Figure S1: Phylogenetic tree of 102 SARS-CoV-2 genome sequences retrieved in this study, and sequences of the Omicron subvariants.
