# Supplementary material for: Evaluation of Multiplex Rapid Antigen Tests for the Simultaneous Detection of SARS-CoV-2 and Influenza A/B Viruses
Source: Biomedicines. 2023 Dec 9;11(12):3267. doi: 10.3390/biomedicines11123267 (PMC10741453; doi:10.3390/biomedicines11123267)
Supplement: Supplementary file 1 [file biomedicines-11-03267-s001.zip › biomedicines-2743123-supplementary.pdf]

biomedicines

Supplementary Materials and Methods

Supplementary Table S1. Mapping statistics of derived sequences of SARS-CoV-2 genomes detected using metagenomics next-generation sequencing

No	Sample ID	Total number of reads for mapping	% of mapped reads	Depth (X)
1	S001	1262808	99.59%	6335.16
2	S002	1769526	99.92%	8877.22
3	S020	2332950	99.99%	11703.76
4	S029	2103966	99.99%	10555.01
5	S007	2092602	99.97%	10498
6	S009	2303542	99.98%	11556.23
7	S022	2406276	100.00%	12071.62
8	S004	2306148	99.98%	11569.3
9	S015	1848050	99.75%	9271.15
10	S021	1695298	99.86%	8504.84
11	S016	1072420	99.64%	5380.03
12	S028	1137960	99.69%	5708.83
13	S093	1961106	99.76%	9838.32
14	S095	706398	99.08%	3543.8
15	S040	822716	99.30%	4127.34
16	S041	1286742	99.40%	6455.23
17	S036	990958	99.86%	4971.36
18	S010	1763922	99.98%	8849.11
19	S097	699676	99.84%	3510.08
20	S043	602732	99.82%	3023.74
21	S098	978836	99.91%	4910.55
22	S023	1495880	99.97%	7504.41
23	S008	1444226	99.95%	7245.28
24	S003	1774240	99.99%	8900.87
25	S019	1318578	99.98%	6614.94
26	S018	1542654	99.99%	7739.07
27	S024	2173792	99.98%	10905.31
28	S099	1697422	99.97%	8515.49
29	S100	2021714	99.99%	10142.38
30	S094	1724248	99.99%	8650.07
31	S012	2112316	99.99%	10596.9
32	S011	1766088	99.99%	8859.97
33	S005	1674104	99.98%	8398.52
34	S027	1746596	99.99%	8762.19
35	S030	1970676	99.99%	9886.33
36	S006	1821814	99.99%	9139.54
37	S047	823868	99.91%	4133.12

Supplementary Table S1. Mapping statistics of derived sequences of SARS-CoV-2 genomes detected using metagenomics next-generation sequencing (Continued)

No	Sample ID	Total number of reads for mapping	% of mapped reads	Depth (X)
38	S045	811622	99.87%	4071.68
39	S059	748974	99.28%	3757.39
40	S068	1209792	99.44%	6069.19
41	S032	2228072	99.99%	11177.62
42	S026	1951070	99.96%	9787.98
43	S025	1732796	99.99%	8692.96
44	S017	2114686	100.00%	10608.79
45	S031	2126324	100.00%	10667.18
46	S014	2172198	99.99%	10897.31
47	S044	2245312	100.00%	11264.11
48	S013	2216882	100.00%	11121.48
49	S033	1875282	99.99%	9407.77
50	S046	1921456	100.00%	9639.41
51	S048	1948906	99.98%	9777.12
52	S089	2101616	99.99%	10543.22
53	S096	2001756	99.93%	10042.25
54	S050	2070496	99.99%	10387.1
55	S067	2297398	100.00%	11525.41
56	S042	2351800	99.98%	11798.33
57	S060	2200202	99.87%	11037.8
58	S071	2290698	99.99%	11491.8
59	S080	1988906	99.64%	9977.79
60	S038	2398256	99.99%	12031.38
61	S052	2427988	100.00%	12180.54
62	S084	2360392	100.00%	11841.43
63	S053	2423404	100.00%	12157.55
64	S069	2248794	99.91%	11281.58
65	S081	2214014	99.99%	11107.09
66	S051	1971620	99.97%	9891.07
67	S058	1722150	99.98%	8639.55
68	S062	2225462	100.00%	11164.53
69	S063	2321708	100.00%	11647.36
70	S076	2257946	99.99%	11327.49
71	S072	2274128	100.00%	11408.67
72	S035	2285694	100.00%	11466.69
73	S087	2114006	100.00%	10605.38
74	S064	2063156	100.00%	10350.28
75	S049	1650150	100.00%	8278.34

Supplementary Table S1. Mapping statistics of derived sequences of SARS-CoV-2 genomes detected using metagenomics next-generation sequencing (Continued)

No	Sample ID	Total number of reads for mapping	% of mapped reads	Depth (X)
76	S073	2252404	100.00%	11299.69
77	S037	2268550	100.00%	11380.69
78	S070	2293278	100.00%	11504.74
79	S085	2213894	100.00%	11106.49
80	S074	2109744	100.00%	10584
81	S078	1756098	100.00%	8809.86
82	S088	1648690	100.00%	8271.02
83	S079	2078980	99.99%	10429.67
84	S077	2324712	100.00%	11662.43
85	S086	2150798	100.00%	10789.96
86	S039	2324064	100.00%	11659.18
87	S075	2437142	100.00%	12226.46
88	S066	2063252	100.00%	10350.76
89	S082	2088568	100.00%	10477.77
90	S083	2016944	100.00%	10118.45
91	S061	1612140	100.00%	8087.66
92	S065	2162310	99.96%	10847.71
93	S090	2053782	99.99%	10303.25
94	S054	2184114	99.91%	10957.09
95	S055	2254472	99.99%	11310.06
96	S091	2120212	99.99%	10636.52
97	S056	2135746	99.99%	10714.44
98	S092	2350204	99.99%	11790.32
99	S057	2358468	99.95%	11831.78
100	S034	2149750	100.00%	10784.7

Supplementary Table S2. Diagnostic accuracy of RAT kits compared with rRT-PCR according to sex

Pathogens	RAT kits	Sex	TP (*n*)	FP (*n*)	TN (*n*)	FN (*n*)	Se (%)	Sp (%)	Kappa
SARS-CoV-2	GenBody	Male	152	0	277	86	63.87	100	0.66
		Female	160	0	212	87	64.78	100	0.63
	InstaView	Male	186	0	277	52	78.15	100	0.79
		Female	203	0	212	44	82.19	100	0.81
	STANDARD Q	Male	172	0	277	66	72.27	100	0.74
		Female	183	0	212	64	74.09	100	0.73
FluA	GenBody	Male	131	0	294	90	59.28	100	0.62
		Female	110	1	279	69	61.45	99.64	0.66
	InstaView	Male	135	0	294	86	61.09	100	0.64
		Female	112	0	280	67	62.57	100	0.67
	STANDARD Q	Male	115	0	294	106	52.04	100	0.55
		Female	84	0	280	95	46.93	100	0.52
FluB	GenBody	Male	8	0	499	8	50	100	0.66
		Female	5	0	449	5	50	100	0.66
	InstaView	Male	8	0	499	8	50	100	0.66
		Female	4	0	449	6	40	100	0.57
	STANDARD Q	Male	4	0	499	12	25	100	0.39
		Female	4	0	449	6	40	100	0.57

Abbreviations: FluA, influenza A virus; FluB, influenza B virus; FP, false positive; FN, false negative; RAT, rapid antigen test; SARS-CoV-2, severe acute respiratory syndrome coronavirus-2; Se, sensitivity; Sp, specificity; TN, true negative; TP, true positive.

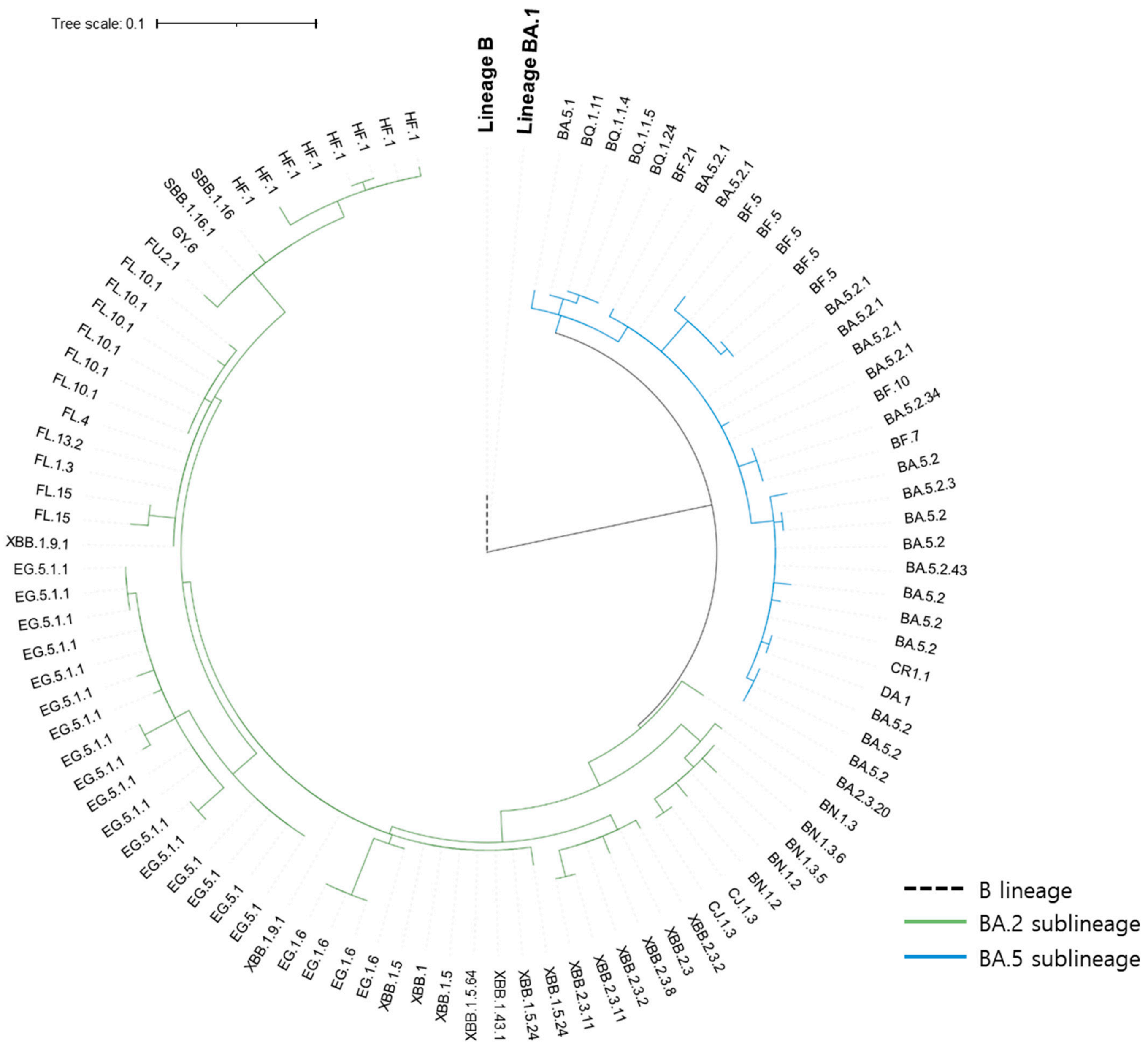

Supplementary Figure S1. Phylogenetic tree of 102 SARS-CoV-2 genome sequences retrieved in this study, and sequences of the Omicron subvariants. Two lineages are marked with different markers (green and blue lines). The B lineage (NCCP-43330) and BA.1 lineage (clinical sample) are marked differently from the other two Omicron sublineages.
